# Martin Peterson: The Ethics of Technology: A Geometric Analysis of Five Moral Principles: Oxford University Press, 2017, 252 pp, USD 74.00 (hbk), ISBN: 9780190652265

**DOI:** 10.1007/s11948-017-0014-0

**Published:** 2018-01-19

**Authors:** Gert-Jan C. Lokhorst

**Affiliations:** Section of Philosophy, Faculty of Technology, Policy and Management, Delft University of Technology, P.O. Box 5015, 2600 GA Delft, The Netherlands

 Peterson discusses five principles (the cost–benefit, precautionary, sustainability, autonomy, and fairness principles) that are used in the ethics of technology.

He analyzes these principles by means of the mathematical theory of Voronoi regions. The concept of a Voronoi region is a simple but intuitively appealing one. Given a finite set of distinct, isolated points in a continuous space, we associate all locations in that space with the closest member of the point set. The result is a partitioning of the space into a set of Voronoi regions. The mathematical theory of Voronoi regions has successfully been applied in many areas, including philosophy (notably by Peter Gärdenfors in his works on “conceptual spaces”). Its application to the ethics of technology seems new.

However, Peterson’s book raises several problems.

First, in his book, “closest” means “most similar in a moral sense.” But how is “most similar in a moral sense” to be understood? As most similar with respect to what? As most similar with respect to catastrophic consequences? As most similar with respect to fairness? Or as most similar with respect to one of the traditional virtues mentioned at https://www.virtuescience.com/the-virtues.html, namely, acceptance, accountability, ambition, assertiveness, beauty, benevolence, bravery, caring, charity, chastity, caution, cleanliness, commitment, compassion, confidence, consideration, contentment, cooperation, courage, courtesy, creativity, curiosity, defiance, dependability, detachment, determination, devotion, diligence, discernment, discretion, discipline, eloquence, empathy, enthusiasm, excellence, faith, faithfulness, flexibility, focus, forbearance, forgiveness, fortitude, friendliness, frugality, generosity, gentleness, grace, gratitude, helpfulness, honesty, honor, hope, humbleness, humility, humor, idealism, integrity, impartiality, industry, innocence, joyfulness, justice, kindness, knowledge, liberality, love, loyalty, magnanimity, majesty, meekness, mercy, moderation, modesty, obedience, openness, orderliness, patience, peace, perseverance, persistence, piety, prudence, punctuality, purity, purposefulness, reliability, resoluteness, resourcefulness, respect, responsibility, restraint, reverence, righteousness, selflessness, self-sacrifice, service, sensitivity, silence, simplicity, sincerity, sobriety, spontaneity, steadfastness, strength, tact, temperance, thankfulness, thrift, tolerance, toughness, tranquility, trust, trustworthiness, truthfulness, understanding, unity, vitality, wisdom, wonder and zeal?

As Kristin Shrader–Frechette writes (*Notre Dame Philosophical Reviews*, 2017.10.30):

Without pre-specified moral-similarity dimensions, each agent likely employs her own implicit dimension(s) to answer Peterson’s moral-similarity request. Thus for the same two cases, one agent might estimate “moral similarity” with respect to catastrophic consequences, while another might estimate similarity with respect to fairness. If so, Peterson has a common moral-similarity label, but no common concept.

Second, Peterson’s geometric construal of domain-specific principles is unclear. He writes (p. 19):

We start with intuitions about a set of nonparadigmatic cases we feel reasonably certain about. The next step is to identify the principles that best account for our intuitions about these cases. Once we have done so, we determine the location of the paradigm cases for these principles ex-post by calculating the mean coordinates of the nonparadigmatic cases we started with.

But he then goes on as follows (p. 19):

**Step 1**

Identify a nonempty set of paradigm cases *C* in which there is no or negligible doubt about what ought to be done and why.

**Step 2**

For each case *c* in *C*, identify the principle *p* that best accounts for the moral analysis of *c*. Let *P* be the set of all such principles.

**Step 3**

Compare all cases, including those not included in *C*, with respect to how similar they are to each other.

**Step 4**

Make a Voronoi tessellation in which each seed point is a paradigm case for some principle *p* in *P*. Each cell of the Voronoi tessellation represents the case covered by *p*, and it is thus the degree of similarity to nearby paradigm cases that determines what it is right or wrong to do in each and every case.

To reconcile these two quotes, “nonparadigmatic” in the first quote should be replaced by “paradigmatic.”

Third, Peterson repeatedly claims that his five principles “are necessary and jointly sufficient for analyzing ethical issues related to new and existing technologies,” but he offers no proof of this claim. Why *five* principles? If five principles partition the moral space into a set of Voronoi regions, then four or six obviously do as well. Why *these* five principles? Why is the maximin principle, for example, not included? Why does Peterson not use some familiar statistical technique, such as factor analysis or principal component analysis, to find out which principles matter?

To me, Peterson’s five principles seem no more than a list of topics that are currently fashionable in the ethics of engineering and that are well-known to students of that field. The list of virtues mentioned at https://www.virtuescience.com/the-virtues.html shows that they are neither *necessary* nor *sufficient*. If somebody left out a principle or added one, then Peterson’s geometric construal would still work, so nothing depends on his claim.

